# Metformin in Colorectal Cancer: Epidemiological Evidence, Predictive Biomarkers, and Implications for Prevention and Treatment

**DOI:** 10.3390/ijms26136040

**Published:** 2025-06-24

**Authors:** Seokho Myung, Youn Young Park, Man S. Kim

**Affiliations:** 1 Translational-Transdisciplinary Research Center, Clinical Research Institute, Kyung Hee University Hospital at Gangdong, College of Medicine, Kyung Hee University, Seoul 05278, Republic of Korea; tjzh13@khu.ac.kr; 2 Department of Medicine, Kyung Hee University College of Medicine, Seoul 02453, Republic of Korea; goodaeng@khu.ac.kr; 3 Department of Surgery, Kyung Hee University Hospital at Gangdong, Kyung Hee University College of Medicine, Seoul 05278, Republic of Korea

**Keywords:** AMPK, biomarkers, colorectal cancer, diabetes mellitus, *KRAS*, MATE1, metformin, patient stratification, personalized medicine, tumor microenvironment

## Abstract

Interest in metformin as a potential anticancer agent for colorectal cancer (CRC) has increased. However, compelling epidemiological links and strong preclinical evidence suggest that metformin has variable efficacy in patients with CRC. This variability highlights the need to identify the patients who are most likely to benefit from effective stratification. We aimed to review the evidence concerning the diverse roles of metformin in CRC prevention and treatment, focusing on identifying and validating the predictive biomarkers essential for selecting patient subgroups that are likely to respond positively. We explored the various molecular pathways through which metformin acts and investigated how these diverse mechanisms might explain the observed differences in patient responses. Epidemiological studies and large meta-analyses have consistently reported reduced CRC incidence and improved survival among patients with diabetes treated with metformin. However, successfully extending these benefits broadly across all patients with CRC or achieving predictable outcomes in advanced disease settings remains a significant challenge. This review consolidates the current knowledge, highlights how different mechanisms interact, critically assesses clinical evidence in light of patient heterogeneity, and advocates for the development and implementation of biomarker-guided personalized therapeutic strategies as key to optimally utilizing the potential of metformin in CRC management. The current challenges and vital future research priorities in this critical area are also outlined.

## 1. Introduction

Colorectal cancer (CRC) is a major cause of cancer-related deaths worldwide and requires novel treatment strategies. Moreover, the association between type 2 diabetes mellitus (T2DM) and a higher risk of developing CRC is well established; individuals with diabetes have an increased incidence compared to those without the disease [1,2]. This connection has sparked investigations into whether common antidiabetic drugs, particularly metformin, offer therapeutic advantages. Metformin, a biguanide, has been a staple in T2DM management for >60 years [3], and it is now being evaluated as an anticancer agent in patients with CRC. However, a robust demonstration of its efficacy presents an ongoing challenge. Beyond its main role in lowering blood sugar levels, metformin possesses anticancer properties in various cancers, including CRC [4,5], reducing the incidence of CRC and improving survival, particularly in patients with diabetes [6,7]. However, metformin’s anticancer effects may extend beyond diabetic populations, although the optimal dosing and patient selection in these patients remain unclear [8,9]. In addition, the degree of benefit varies considerably among individuals and those at different disease stages or sexes, clearly indicating the need to accurately identify the patients most likely to respond positively. Thus, identifying and validating predictive biomarkers is both an incremental step and fundamental to unlocking the full potential of metformin in CRC therapy. This comprehensive review aims to critically assess the current understanding of the role of metformin in CRC by focusing on patient heterogeneity and predictive biomarkers. The objectives of this study are as follows:

- Identifying and evaluating promising predictive biomarkers that are vital for stratifying patients and forecasting metformin responses in CRC are indispensable tools for future clinical use.
- Analyzing the complex and varied molecular pathways through which metformin exerts its anticancer effects and exploring how understanding these mechanisms, alongside biomarkers, might shed light on the differing patient outcomes.
- Analyze and integrate clinical evidence from epidemiological and intervention studies, specifically considering the variation in observed results as the rationale for adopting biomarker-guided approaches.
- To investigate the effect of metformin on the tumor microenvironment (TME) and discuss how relevant characteristics might interact with biomarkers to influence treatment response.
- To emphasize the implications of the current findings for developing biomarker-based personalized therapeutic strategies as the key to optimizing the use of metformin in CRC.
- To highlight current hurdles and future research priorities in this field, particularly regarding biomarker validation and clinical practice.

## 2. Biomarkers for Metformin Response in CRC: Essential Tools for Patient Stratification

Given the observed variations in patient response to metformin, identifying reliable biomarkers is essential for effective patient stratification and successful integration of metformin into personalized CRC therapy. These biomarkers can help identify individuals or tumor subtypes that are most likely to gain meaningful clinical benefits from a drug, thereby optimizing treatment results and preventing unnecessary exposure in those who do not respond positively. Several factors, including genetic alterations, protein expression levels, and metabolic profiles, are currently being investigated for their roles as potential predictive biomarkers (Table 1).

### 2.1. KRAS Mutation Status as a Key Predictive Biomarker

Genetic changes are fundamental in determining cancer behavior and drug responses. Among these, *KRAS* mutation status has emerged as a particularly important predictive biomarker in patients with CRC. Xie et al. [10] reported that the presence of a *KRAS* mutation is a critical factor for metformin responsiveness in CRC. Their findings indicated that patients with metastatic CRC and *KRAS* mutations and co-occurring diabetes who received metformin experienced a significantly longer median survival (an additional 37.8 months) than those treated with other blood sugar-lowering drugs alongside standard systemic therapy. In contrast, metformin did not provide a significant survival advantage in patients with wild-type *KRAS* tumors or T2DM. This selective and substantial benefit observed specifically in *KRAS*-mutant tumors highlights *KRAS* mutation as a clinically validated and significant predictive marker, suggesting that patients with this genetic modification represent a prime group for metformin treatment. This difference in response appears to be linked, at least in part, to how the drug transporters essential for metformin uptake and accumulation behave differently in the presence of *KRAS* mutations.

### 2.2. Drug Transporter Expression as Determinants of Metformin Accumulation

**Figure 1:**
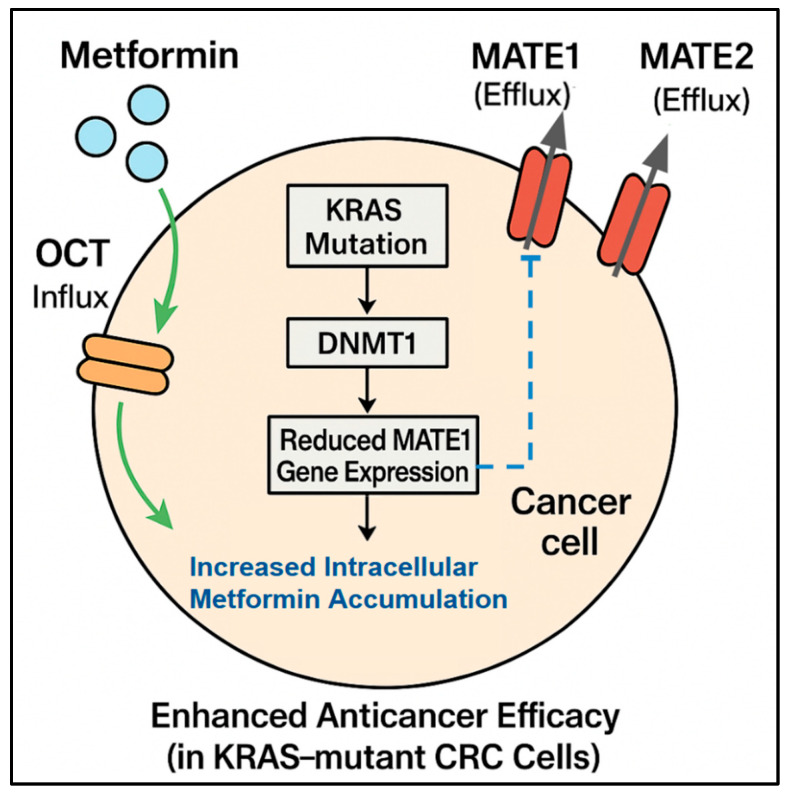
Influence of biomarkers on metformin accumulation and efficacy: This diagram shows a CRC cell and illustrates how specific biomarkers, particularly *KRAS* mutation status and drug transporter expression, determine the intracellular accumulation and efficacy of metformin. Metformin enters the cell via influx transporters (OCTs) and exits via efflux transporters (MATE1, MATE2). A *KRAS* mutation can lead to *DNMT1*-mediated silencing and reduced *MATE1* gene expression, resulting in reduced MATE1 efflux. This decreased efflux causes increased intracellular metformin accumulation/concentration, which in turn leads to enhanced anticancer efficacy, specifically observed in *KRAS*-mutant CRC cells.

Metformin uptake into cells and exit processes necessary for its action inside the cell rely heavily on the function and amount of various organic cation transporters (OCTs) for uptake and multidrug and toxic compound extrusion (MATE) transporters for efflux [11,16]. Different expression levels of these transporters can lead to significant variations in the extent of metformin build-up within tumor cells (Figure 1). This accumulation level is a key factor in determining the drug concentration inside the cell and consequently influences therapeutic effectiveness and patient response. Therefore, the expression patterns of these transporters may serve as valuable predictive biomarkers.

#### 2.2.1. MATE1 Expression Predicts Response

The expression of MATE1 appears to be a key negative predictive biomarker of metformin efficacy. Xie et al. [10] reported an association between *KRAS* mutation status and MATE1 expression, which helps explain the differential responses. They found that *KRAS*-mutant tumors exhibited silenced MATE1 expression. Silencing occurs because DNA methyltransferase 1 (DNMT1) causes hypermethylation of the MATE1 promoter, leading to reduced metformin efflux from cells. This indicates that more metformin accumulates inside *KRAS*-mutant cells. This mechanistic insight provides a molecular reason for the selective vulnerability of *KRAS*-mutant CRC to metformin and strongly suggests that low MATE1 expression levels are a promising predictive biomarker, particularly alongside *KRAS* status, for identifying patients who can accumulate sufficient intracellular metformin to achieve therapeutic effects (Figure 1).

Recent clinical studies have reinforced MATE1’s critical role in determining metformin pharmacokinetics and its potential interactions with other medications. Guchelaar et al. [16] conducted a crossover pharmacokinetic study to examine the interactions among trifluridine (used in metastatic CRC treatment), metformin, and cimetidine (OCT2/MATE1 inhibitor). These findings highlight the importance of considering drug transporter profiles not only as biomarkers for how well metformin works but also for predicting potential drug-drug interactions in patients with cancer receiving combination treatments.

#### 2.2.2. OCT and MATE2 Transporter Profiles Influence Sensitivity

In addition to MATE1, other transporters play a role in controlling intracellular metformin levels and influencing the response, potentially acting as components of transporter-based biomarker panels. Chowdhury et al. [11] showed that higher MATE2 expression was correlated with resistance to the effects of metformin on cell growth in cancer cell lines, suggesting that MATE2 is another potential negative predictive biomarker. Conversely, OCT3 expression was associated with increased sensitivity to a metformin-induced reduction in oxygen consumption. These results highlight the complex interplay between different drug transporters in determining the final cellular response to metformin and emphasize that checking a wider set of transporter expression levels, perhaps alongside genetic markers such as *KRAS*, may be useful for implementing more accurate and comprehensive predictive biomarker strategies.

### 2.3. Adenosine Monophosphate-Activated Protein Kinase Pathway Components as Indicators of Sensitivity

The primary molecular target of metformin is the adenosine monophosphate-activated protein kinase (AMPK) pathway. The state or activity of the components of this pathway can serve as predictive biomarkers. The baseline activity or specific parts of this pathway can affect the sensitivity of cells to metformin. Morrison et al. [12] reported that elevated basal AMPK activity, specifically of the AMPKα1 catalytic subunit, makes CRC cells more susceptible to metformin-induced growth inhibition. CRC cell lines with naturally high AMPKα1 activity showed greater vulnerability to metformin’s effects, suggesting that pre-existing high AMPK status, or markers reflecting its activity, could be a potential predictive biomarker for stratifying patients and identifying those likely to respond positively to metformin’s activation of AMPK.

Ye et al. [17] highlighted the role of AMPK activation in CRC, particularly when *KRAS* mutations are present. They demonstrated that AMPK activation could overcome resistance to anti-EGFR antibodies owing to *KRAS* mutations in CRC. This finding confirms a mechanistic link between *KRAS* mutation status, AMPK pathway activity, and treatment response, further supporting the importance of considering both genetic changes and metabolic pathway status when predicting the effectiveness of metformin.

### 2.4. Metabolic Signatures as Predictors of Response and Resistance

Cancer cells often have altered metabolic profiles that support their rapid growth, and these unique metabolic features can influence their reactions to drugs that target their metabolism, such as metformin. Identifying these metabolic dependencies or weaknesses can lead to the discovery of new and important predictive biomarkers, as well as markers of resistance that might be overcome with combination therapies.

#### 2.4.1. Glutamine Metabolism and Metformin Sensitivity

Changes in glutamine metabolism are associated with metformin sensitivity and can act as predictive markers or indicate combination therapies. Kim et al. [13] reported that the effects of metformin on cancer stem cells depend on glutamine metabolism. They observed that CRC cells that were highly dependent on glutamine were resistant to metformin treatment. This resistance can be overcome by either removing glutamine from the cell culture medium or simultaneously treating cells with glutaminase inhibitors. This suggests that markers of glutamine dependence can predict metformin resistance in certain CRC subtypes and can be used to identify patients who may benefit from combination therapy targeting glutamine metabolism to boost the effectiveness of metformin. Lee et al. [18] further supported the value of combining metabolic inhibitors when treating patients with CRC. Their research showed that combining AMPK activators such as phenformin (a biguanide similar to metformin) with metabolic inhibitors such as 2-deoxy-D-glucose (2DG) produced synergistic antitumor effects in colon tumors, particularly in those with *BRAF* and p53 mutations. This combination effectively slowed tumor growth through various mechanisms, including cell cycle arrest, apoptosis, and autophagy, highlighting how metabolic profiling and mutational status together could guide the best combination strategies involving metformin or related compounds.

#### 2.4.2. Transcriptomic Profiles for Identifying Responsive Subtypes

Recent studies examining gene expression patterns (transcriptomics) have offered insights into the broader metabolic and molecular landscape associated with diabetes-driven CRC progression and the potential response to metformin. This development opens new avenues for the identification of new predictive biomarkers based on how genes are expressed [14]. Global gene expression analysis can reveal novel metabolic or signaling patterns that may serve as predictive biomarkers for identifying responsive subgroups. Luo et al. [14] used transcriptomics to investigate the molecular mechanisms linking T2DM and CRC progression and identified key genes, such as *COX11*, whose expression may correlate with outcomes or treatment response. Such studies exploring extensive gene expression profiles could uncover new metabolic or signaling signatures that are useful for predicting which patients are most likely to benefit from metformin therapy. Furthermore, new gene signatures revealing distinctive immune-metabolic changes across different tumor types, including CRC, have been proposed to be associated with metformin responsiveness [15]. This finding further highlights the complex interactions between immune and metabolic factors in the TME as possible sources of biomarkers for stratifying patients.

### 2.5. Biomarker Behavior in Diabetic Versus Non-Diabetic Populations: Implications for Patient Stratification

The effectiveness of biomarkers in predicting metformin response may vary significantly between diabetic and non-diabetic populations, necessitating population-specific validation and potentially different biomarker thresholds. This variation stems from fundamental differences in the baseline metabolic states, insulin sensitivity, and cellular signaling pathways between these populations.

In patients with diabetes, chronic hyperglycemia and insulin resistance create a distinct metabolic milieu that may influence biomarker expression and drug transporter functions. For instance, MATE1 expression and function may be altered in patients with diabetes owing to chronic exposure to high glucose levels and inflammatory mediators [16]. Similarly, baseline AMPK activity, a critical determinant of metformin sensitivity, is often impaired in patients with diabetes owing to persistent metabolic stress, potentially affecting the predictive value of AMPK-related biomarkers [12].

Patients without DM present different challenges in the interpretation of biomarkers. These patients may lack the chronic metabolic dysfunction observed in diabetes, but they also lack the established metabolic vulnerability that makes them more responsive to the effects of metformin. Traditional biomarkers, such as KRAS mutation status, in patients with non-diabetic CRC may have different predictive values compared with diabetic cohorts [9]. Additionally, optimal metformin dosing in patients without diabetes may require different biomarker-guided approaches, as the drug mechanisms in normoglycemic individuals may involve alternative pathways beyond traditional glucose metabolism modulation.

Metabolic signatures may be particularly affected by the diabetes status. Glutamine metabolism dependence, identified as a mechanism of metformin resistance, may differ between populations owing to baseline differences in amino acid metabolism and cellular energy states [13]. Furthermore, transcriptomic profiles and immune-metabolic signatures may reflect population-specific patterns, which require separate validation cohorts for optimal clinical utility [15]. These findings underscore the critical need for the development and validation of population-stratified biomarkers. Future studies should specifically address whether biomarker panels require different compositions, cut-off values, or interpretation algorithms when applied to patients with and without diabetes. Such population-specific approaches are essential for implementing personalized metformin therapy across diverse patient populations.

## 3. Molecular Mechanisms of Metformin Action: The Basis for a Differential Response

**Figure 2:**
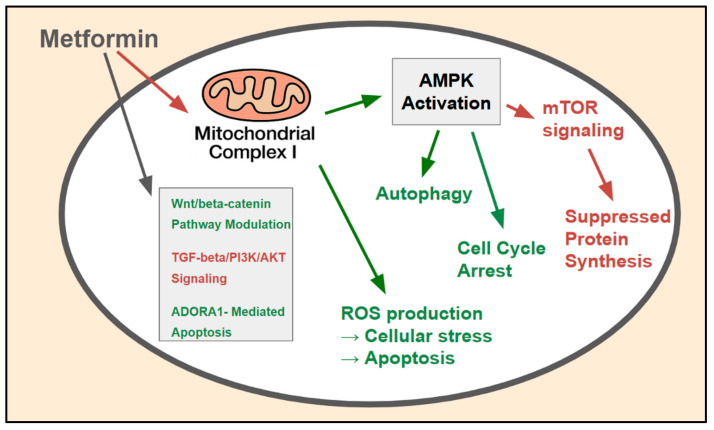
AMPK-dependent and -independent molecular mechanisms of metformin action in CRC cells: The major molecular pathways through which metformin exerts its anticancer effects within CRC cells. The primary mechanism involves inhibition (red) of mitochondrial complex I, leading to AMPK activation (green), which subsequently inhibits (red) the mTOR pathway, suppresses (red) protein synthesis, induces (green) cell cycle arrest, and modulates (green_ autophagy. AMPK-independent mechanisms include ROS production and the modulation of key oncogenic signaling pathways such as the Wnt/beta-catenin, TGF-beta/PI3K/AKT, and *ADORA1*-mediated apoptosis pathways.

Metformin exerts its anticancer effects through a variety of pathways, depending on the target AMPK and other pathways operating independently (Figure 2) [19]. Understanding these diverse mechanisms is important not only for designing effective combination therapies but also for interpreting how different CRC subtypes, as defined by biomarkers, might react to metformin based on which pathways are active or suppressed. These mechanisms often work together to slow down the growth, proliferation, and survival of cancer cells.

### 3.1. AMPK-Dependent Pathways: A Primary Biomarker-Influenced Mechanism

AMPK activation is the most extensively studied mechanism of action of metformin. AMPK is a key enzyme that acts as a central regulator of energy in CRC [20]. Metformin activates AMPK mainly by inhibiting mitochondrial complex I (discussed below), which leads to an increased AMP:ATP ratio. Once activated, AMPK phosphorylates various downstream targets, ultimately slowing down anabolic processes and accelerating catabolic ones. Bozzi et al. [21] showed that metformin temporarily slowed CRC cell growth through AMPK activation, which leads to several downstream effects that contribute to the anticancer activity of metformin.

AMPK-mediated phosphorylation of TSC2 results in negative regulation of mTORC1, leading to the suppression of mTOR signaling, a critical pathway governing protein synthesis, cellular growth, and proliferation [22]. This is considered the primary mechanism through which metformin curbs cancer cell growth, and the state of the mTOR pathway or its regulators may influence this effect in the following manner:

1. Suppression of protein synthesis: Following mTOR inhibition, protein synthesis is reduced, limiting the availability of building materials for rapidly dividing cancer cells [22];
2. Cell cycle arrest: Metformin-induced AMPK activation can result in a greater number of cyclin-dependent kinase inhibitors and fewer cyclins, causing the cell cycle to pause, typically in the G0/G1 phase, and thus preventing uncontrolled cell division [23]. Thus, proteins that regulate the cell cycle may serve as potential pharmacodynamic biomarkers;
3. Induction of autophagy: AMPK triggers autophagy, a cellular process that removes damaged organelles and proteins [24]. Autophagy can facilitate cancer cell survival. However, in certain situations, it can contribute to the toxic effects of metformin or support cell survival under drug-induced metabolic stress. The context-dependent role of autophagy in the effects of metformin on CRC requires further study, and markers of autophagic flux should be explored as potential biomarkers.

Lee et al. [25] provided further insights into the role of metformin in CRC cells resistant to tumor necrosis factor-related apoptosis-inducing ligands (TRAIL). The authors showed that metformin reduced the inhibitory effect of an X-linked inhibitor of apoptosis by blocking the AKT and NF-κB pathways while activating the C/EBP homologous protein via endoplasmic reticulum stress. These findings point to other potential biomarkers related to components of the cell death pathway that may predict the response to metformin, especially when combined with TRAIL therapy [25].

### 3.2. AMPK-Independent Mechanisms: Contributing to Variable Responses

Although AMPK is a primary mediator of the effects of metformin, it also exhibits anticancer activity through pathways that do not rely on AMPK activation, particularly at higher concentrations, which can reach certain tissues such as the colon or specific cellular settings [26]. These alternative mechanisms add to the complex nature of metformin’s action and can lead to different responses depending on the molecular makeup of the tumor.

#### 3.2.1. Reactive Oxygen Species Production: A Context-Dependent Effect

Metformin triggers the production of reactive oxygen species (ROS) [27]. High ROS levels can damage cells and cause cell death, whereas a moderate increase can activate stress pathways that contribute to cell cycle arrest and apoptosis. This effect seems particularly relevant in cancer cells because of their high metabolic activity and frequent oxidative stress, making them more vulnerable to further ROS insults than normal cells [28]. The balance between ROS induction and the capacity of cells to handle antioxidants, which varies among tumors, may influence metformin sensitivity and can potentially be evaluated using biomarkers.

Chuang et al. [29] showed that combining metformin with curcumin significantly boosted ROS production in CRC cell lines by activating the NRF2/KEAP1 protein pathway. They observed varying responses to *NRF2* activation in different CRC cell lines (CT26 and HCT116). This suggests that differences in how oxidative stress responses are regulated could contribute to variable sensitivity to metformin-based treatments and that markers of the cell’s redox balance might serve as predictive biomarkers.

#### 3.2.2. Mitochondrial Complex I Inhibition: A Fundamental Target with Downstream Implications

A key and possibly the most direct target of metformin is the mitochondrial complex I within the electron transport chain [30]. Metformin impairs mitochondrial respiration and reduces ATP production by inhibiting complex I activity. This causes an increase in the AMP:ATP ratio, which activates AMPK. However, the direct inhibition of complex I, even separate from AMPK activation, can induce energy stress responses and potentially contribute to ROS production. This affects highly proliferative cancer cells that rely heavily on mitochondrial oxidative phosphorylation for energy production [31]. A tumor’s metabolic state and whether it depends more on oxidative phosphorylation or glycolysis (the Warburg effect) could influence sensitivity to metformin and potentially represent metabolic biomarkers.

### 3.3. Modulation of Oncogenic Signaling Pathways: Intersecting with Biomarkers

In addition to metformin’s effects on the core AMPK pathway and direct metabolism, it influences various signaling pathways crucial for the start, progression, and spread of various cancers, including CRC [32,33,34,35,36,37,38]. Interference with these pathways adds to the broader anticancer effects of metformin and may also explain why responses differ depending on tumor mutations and active pathways, highlighting the importance of biomarkers.

#### 3.3.1. Wnt/Beta-Catenin Pathway Modulation

The Wnt/beta-catenin pathway is a fundamental signaling cascade involved in cell growth, specialization, and stem cell maintenance. Its abnormal activation is often seen in CRC, frequently owing to mutations in APC or beta-catenin [33]. Zhang et al. [34] showed that metformin reduced cancer cell stemness and inhibited epithelial-mesenchymal transition (EMT), a process vital for metastasis, through suppressing the Wnt3a/beta-catenin signaling pathway. This indicates that metformin can counteract key processes driving CRC progression by modulating this critical pathway, potentially offering a therapeutic benefit for tumors with activated Wnt signaling. The mutational status of APC or beta-catenin, or the activation state of the Wnt pathway, could potentially serve as biomarkers for predicting response to this aspect of metformin’s action.

#### 3.3.2. TGF-Beta/PI3K/AKT Signaling Inhibition

The TGF-beta signaling pathway plays a complex role in cancer, often acting to suppress tumors in early stages but promoting invasion and metastasis later [35]. The PI3K/AKT pathway is a major signaling pathway that promotes cell survival. It is frequently activated in CRC and is involved in cell growth, proliferation, and survival [36]. Xiao et al. [37] identified *INHBA* as a target through which metformin inhibits the TGF-beta/PI3K/AKT signaling axis. This inhibition led to reduced CRC growth and metastasis, highlighting the ability of metformin to interfere with interconnected pathways that drive aggressive tumor behavior. The activation state or mutational profile of components within the TGF-beta and PI3K/AKT pathways could potentially affect sensitivity to metformin and act as predictive biomarkers.

#### 3.3.3. Adenosine A1 Receptor-Mediated Apoptosis

Metformin also triggers apoptosis, or programmed cell death, facilitating the elimination of cancer cells via mechanisms involving adenosine receptors. Lan et al. [38] reported that metformin induces apoptosis in CRC cells by increasing adenosine A1 receptor (*ADORA1*) levels. This presents another distinct mechanism through which metformin can directly cause cancer cell death, independent of its metabolic effects, and suggests that *ADORA1* expression could potentially influence the response to treatment and could be explored as a biomarker.

## 4. Clinical Evidence: Highlighting the Need for Patient Stratification

Epidemiological research initially observed a potential link between metformin use and reduced incidence of cancer among individuals with diabetes [39,40]; however, subsequent studies have raised important concerns regarding methodological limitations, including the retrospective nature of most analyses and susceptibility to selection biases [41,42]. Despite these limitations, a substantial body of preclinical evidence supports the direct anticancer effects of metformin, particularly through the suppression of mitochondrial oxidative phosphorylation and induction of energetic stress in tumors with specific molecular vulnerabilities, such as LKB1 loss or deficiencies in mitochondrial complex I [43,44,45]. These mechanistic insights provide the rationale for ongoing clinical trials investigating metformin as a potential adjunct in cancer therapy [46,47]. Although observational studies and meta-analyses have provided encouraging clinical signals [7], variations in outcomes across studies and patient groups underscore the need for effective patient stratification based on predictive biomarkers to ensure consistent and meaningful clinical benefits (Table 2).

### 4.1. Meta-Analyses and Systematic Reviews: Evidence for the Association Between Metformin and CRC and the Need for Further Precision

Multiple large-scale meta-analyses and systematic reviews have consistently demonstrated a protective link between metformin use and CRC outcomes, particularly in patients with diabetes. Wang et al. [6] reported a significant reduction in the incidence of both CRC and adenoma formation in metformin users. Their meta-analysis also indicated improved overall survival and CRC-specific survival in patients with diabetes who were taking metformin. Sex-specific differences in survival benefits were identified, with women showing a greater reduction in CRC-specific mortality (hazard ratio [HR], 0.63) than men (HR 0.84) [6]. Another comprehensive systematic review and meta-regression analysis by Ng et al. [7] similarly concluded that metformin use was associated with a significant reduction in the incidence of colorectal adenoma and cancer, and an improvement in CRC outcomes, reinforcing the findings of other large analyses.

A recent meta-analysis by Xiang et al. [50] further broadened our understanding of the association between metformin and various cancer types using Mendelian randomization combined with database analyses. This meta-analysis provides robust evidence supporting the protective effect of metformin against CRC while highlighting the differing effects across various cancer types, further stressing the need for cancer- and patient-specific approaches to metformin therapy.

Although these studies provide strong epidemiological evidence for an association between metformin use and CRC and suggest beneficial effects, particularly in populations with diabetes, they also point to variability in the magnitude of the observed effects across different groups and study designs. This heterogeneity in the findings strongly implies that not all patients with CRC benefit equally, emphasizing the need to identify predictive biomarkers to select those who are most likely to respond positively. The significant heterogeneity observed across studies indicates that important patient subgroups are being masked, highlighting the urgent need for biomarker-guided patient stratification [6,7].

### 4.2. Nationwide Cohort Studies: Confirming Associations, Limited by Confounding

Numerous nationwide cohort studies have provided robust epidemiological support for the association between metformin use and improved CRC outcomes. In 2020, Huang et al. [48] showed that metformin use after CRC diagnosis was associated with a significant reduction in both overall and CRC-specific mortality in patients with diabetes. This finding is particularly significant, as it suggests that metformin may have therapeutic potential not only in preventing CRC but also in individuals already diagnosed with CRC.

However, these observational studies, which are powerful tools for identifying associations in large groups, are susceptible to confounding factors and biases (such as immortal time bias) that can influence outcomes and contribute to variability in findings. This makes it challenging to draw definitive conclusions concerning efficacy across all subgroups without biomarker validation. The positive indications from these studies, when viewed alongside the variability in response to treatment, further highlight the need for clinical trials guided by biomarkers to confirm the effectiveness of metformin in patient populations defined before starting a trial. These observational studies are limited by immortal time bias and unmeasured confounding factors, emphasizing the need for randomized trials with biomarker stratification.

### 4.3. Clinical Trials: Variable Outcomes Highlight the Need for Stratification

Although epidemiological data are compelling, prospective randomized controlled trials are the gold standard for definitively proving therapeutic effectiveness, and importantly, for identifying which patients benefit the most. Recent clinical trials have investigated the potential of metformin, often in combination with other treatments, in controlled environments. For instance, Akce et al. [49] conducted a Phase II trial investigating the combination of nivolumab (an immune checkpoint inhibitor) and metformin in patients with microsatellite-stable metastatic CRC who did not respond to previous treatments. The clinical efficacy in this trial was limited; however, correlative studies have shown an increase in the number of immune cells infiltrating the tumor, suggesting that metformin may influence the immune system, even in this setting.

In a recent multicenter randomized trial with a factorial design by Brown et al. [51], the effects of exercise and metformin on myokine concentrations in patients with breast cancer and CRC were examined. The findings of this study provide insights into how metformin affects the cytokines produced by muscles (myokines), potentially mediating some of its beneficial effects on cancer outcomes. These findings suggested that myokine profiles could potentially serve as pharmacodynamic biomarkers of metformin activity.

The limited clinical response observed in these trials, despite indications from preclinical studies, highlights the complexity of the effects of metformin on advanced disease and emphasizes the need for targeted patient selection using predictive biomarkers. The results of ongoing and future clinical trials, particularly those specifically designed to evaluate efficacy in patient subgroups defined by biomarkers, are key to establishing the optimal role of metformin and identifying the correct patient population for CRC treatment plans. Limited clinical trial efficacy highlights critical gaps such as inadequate patient selection, suboptimal dosing, and insufficient understanding of combination strategies, all pointing to the need for biomarker-guided trial designs [49].

## 5. Metformin and the TME: A Source of Biomarkers and Potential Therapeutic Targets

The TME is a complex system that plays an important role in tumor growth, spread, and response to treatment. Metformin affects various parts of the TME, contributing to its overall anticancer effects and potentially influencing why patients respond differently [52]. The characteristics of the TME, or how metformin changes it, can also potentially serve as biomarkers for predicting response or identifying targets for combination therapy.

### 5.1. Immune Modulation Within the TME

**Figure 3:**
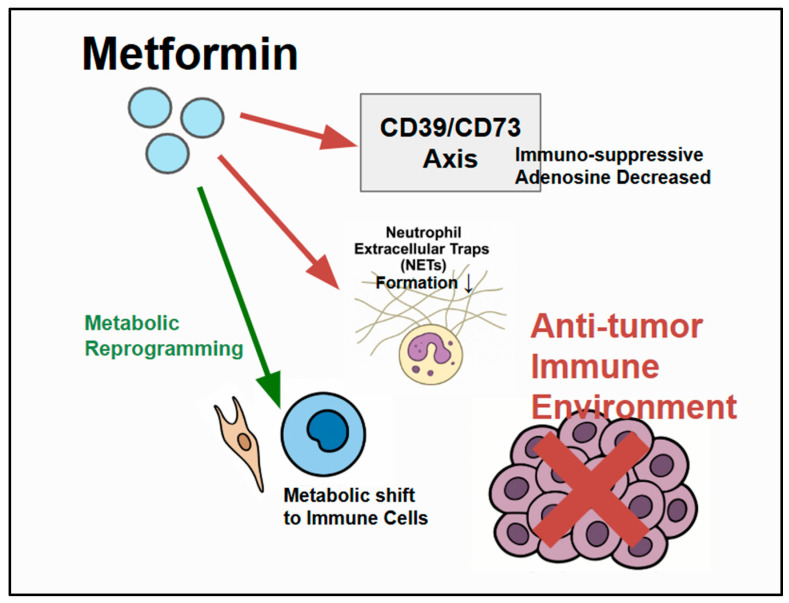
Metformin’s modulation of the TME: Metformin influences components of the CRC TME by interacting with cancer cells, immune cells (neutrophils, T cells), and stromal cells. Metformin has specific immune-modulatory effects, including the inhibition (red) of neutrophil extracellular trap (NET) formation. The CD39/CD73 axis generates immunosuppressive adenosine, which suppresses antitumor immunity, and metformin can modulate/counteract (red) this pathway to potentially enhance antitumor immunity. Metformin also influences metabolic reprogramming within the TME. Enhanced (green) antitumor immunity can then inhibit CRC cells.

Metformin can alter the immune landscape within the TME, potentially shifting the balance towards an antitumor immune response (Figure 3) [53]. The modulation of immune cells and their interactions within the tumor may be a key part of the therapeutic effect of metformin. Variations in the TME immune composition or the effect of metformin on immune cells may influence treatment outcomes and serve as predictive biomarkers.

#### 5.1.1. Neutrophil Extracellular Traps and Metformin’s Influence

Neutrophil extracellular traps (NETs) are web-like structures released by neutrophils. They promote cancer progression and metastasis by trapping circulating tumor cells and creating an environment that encourages their spread. Chen et al. [54] demonstrated that metformin may improve outcomes in patients with diabetes-related CRC, partly by reducing NET formation. This suggests that one of the benefits of metformin is its ability to dampen the inflammatory processes driven by neutrophils, which stimulate tumor growth. The number of NETs in the TME could potentially be a biomarker for predicting metformin response, and a reduction in NETs could be a pharmacodynamic biomarker for metformin.

Liu et al. [55] reported an additional effect of metformin on the immune system. They showed that metformin inhibited the expression of the *NLRP3* inflammasome and regulated the inflammatory environment in CRC. The *NLRP3* inflammasome plays a key role in inflammatory responses, and its inhibition may contribute to slowing CRC progression. This mechanism adds to the understanding of how metformin affects inflammation in the TME and suggests that markers of inflammasome activation could potentially serve as predictive or pharmacodynamic biomarkers.

#### 5.1.2. CD39/CD73 Axis Modulation and Immune Evasion

The CD39/CD73 ectonucleotidase axis regulates extracellular adenosine levels in the TME. Adenosine, which is often produced in tumor environments with low oxygen and metabolic stress, is a strong immunosuppressant that can inhibit the activity of various antitumor immune cells, such as T and NK cells [56]. Roliano et al. [57] reviewed CRC and purinergic signaling and highlighted the key role of the CD39-CD73 axis in promoting immune suppression and CRC progression within the TME. Metformin influences this pathway, and combining metformin with drugs that block CD39 boosts antitumor immunity in laboratory models [58]. This suggests that metformin may counteract adenosine-driven immune suppression within the TME, potentially enhancing the effectiveness of immunotherapy. Therefore, the status of the CD39/CD73 axis, levels of extracellular adenosine, and the presence of immune cells infiltrating a patient’s tumor may be potential biomarkers for predicting the effects of metformin on the immune system and the overall response, especially when used with immunotherapy.

### 5.2. Metabolic Reprogramming in the TME: Implications for Response

The effect of metformin on metabolism is not limited to cancer cells; it also extends to other cells in the TME, including immune cells and stromal fibroblasts [45]. Through its effect on cellular energy metabolism, metformin can create a microenvironment that is less suitable for tumor growth and potentially improve the function of antitumor immune cells. For example, altering the availability of metabolic building blocks or the build-up of metabolic byproducts can influence the activation and function of immune cells [59]. This metabolic reshaping within the TME is complex, and more research is needed to fully determine how metformin orchestrates these changes and how the metabolic characteristics of the TME influence treatment outcomes and differential patient responses. Metabolic profiling of the TME may reveal new biomarkers for predicting a patient’s response to metformin treatment.

## 6. Implications for Personalized Medicine: Biomarkers as a Foundation

**Figure 4:**
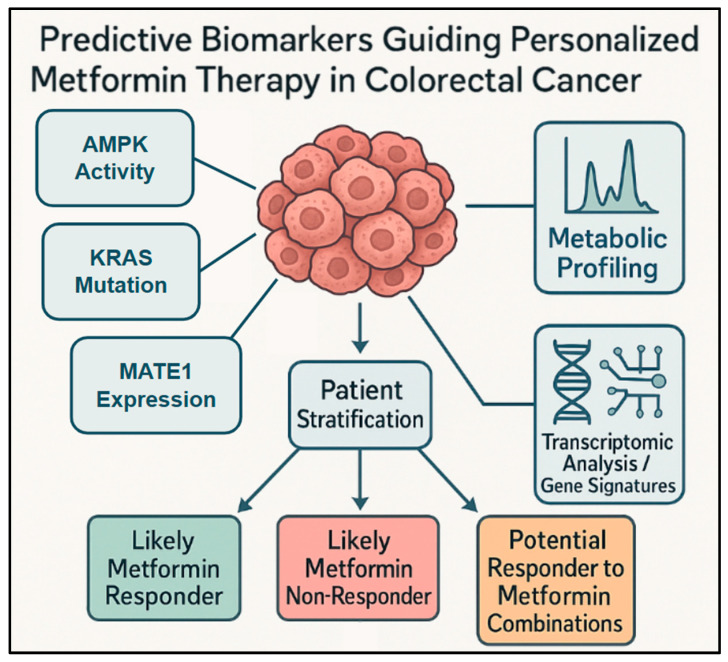
Diverse and emerging biomarkers for metformin response in CRC: This conceptual diagram presents a broader view of diverse and emerging predictive biomarkers used for stratifying patients with CRC for metformin therapy. It shows how analyzing different types of biomarkers from the tumor or patient, such as AMPK activity, metabolic profiling, and transcriptomic analysis/gene signatures (including immune-metabolic profiles), contributes to patient stratification. This stratification process helps to identify patient groups such as likely metformin responders, likely metformin non-responders, or potential responders to metformin combination, ultimately guiding biomarker-guided personalized metformin therapy.

Considering the variable clinical responses to metformin in CRC and the growing evidence that specific biomarkers influence these responses, the development and implementation of personalized treatment approaches guided by biomarkers are essential for effectively managing CRC with metformin (Figure 4) [60]. By accurately sorting patients according to how likely they are to respond positively, clinicians can make better treatment choices, maximize the benefits of metformin for those who are likely to respond positively, and avoid unnecessary administration of the drug to non-responders, which could delay effective treatment.

### 6.1. Biomarker-Guided Treatment Selection: The Path Forward

The promising predictive biomarkers discussed in Section 2 have a significant potential to guide the use of metformin in CRC and represent a clear direction for realizing its full potential. Incorporating these biomarkers into routine clinical decision-making, once validated in prospective trials, can help identify patient groups that are most likely to achieve clinical benefits.

- Patients with *KRAS*-mutant CRC: these patients, particularly those with diabetes, appear to significantly benefit from metformin [10]. Testing for *KRAS* has already been performed in metastatic CRC to determine eligibility for anti-EGFR monoclonal antibody therapies, such as cetuximab or panitumumab, making it a readily usable predictive marker.
- Tumors with low MATE1 expression: low levels of MATE1 are correlated with higher metformin accumulation inside cells and greater sensitivity [10,16]. Assessing MATE1 expression could help refine patient selection even within the *KRAS*-mutant group.
- High basal AMPK activity: Cancer cells with high AMPK activity might be more vulnerable to the effects of metformin on tumor growth [12,17]. Developing reliable and standard tests to measure tumor AMPK activity could help identify responsive patients.
- Specific metabolic or immune-metabolic signatures: analysis of gene expression patterns (transcriptomics) and metabolic profiles of tumor tissues or blood samples can identify new and complex biomarkers for response [14,15]. Further research is required to validate these complex signatures as predictive markers in the clinical setting.

Integrating these biomarkers into clinical practice through well-designed prospective trials is a critical next step in confirming their usefulness in guiding patient selection for metformin therapy and moving towards a more targeted and personalized approach.

### 6.2. Combination Strategies Guided by Molecular and Biomarker Profiles

The favorable safety profile and varied mechanisms of action of metformin make it an appealing candidate for appropriately targeted combination therapies aimed at boosting its effectiveness and overcoming resistance to other treatments [60]. Combining metformin with other agents, guided by a tumor’s molecular characteristics and identified biomarkers, could lead to synergistic effects and improved outcomes, especially in patients who are less likely to respond to metformin alone or in those whose tumors have specific resistance mechanisms that can be targeted by combination partners. Potential combination strategies include the following:

- Metformin may increase the sensitivity of cancer cells to traditional chemotherapeutic drugs by altering cell metabolism or by affecting survival pathways. This could potentially allow for lower doses or help overcome resistance, particularly in tumors with specific metabolic characteristics that have been identified [61].
- The effects of metformin on the immune system, such as reducing NETs and influencing the CD39/CD73 axis, suggest that metformin could work well in immunotherapies. This could help prevent the TME from becoming immunosuppressive and boost the effects of the immune checkpoint blockade [49,53]. Biomarkers related to the tumor immune microenvironment and immune checkpoint pathways can guide these combinations.
- Targeted therapies: combining metformin with drugs targeting specific molecular changes in a tumor could lead to more effective and lasting responses by simultaneously targeting multiple pathways essential for tumor growth and survival [62]. Testing for targetable changes by using biomarkers is fundamental to this approach.
- With metabolic inhibitors, as shown by Lee et al. [18], combining metformin or related biguanides with other metabolic inhibitors, such as 2-deoxy-D-glucose (2DG), can produce synergistic antitumor effects, particularly in tumors with specific genetic changes such as *BRAF* and *p53* mutations. These genetic markers can guide patient selection using combined approaches.

Designing rational combination regimens based on a thorough understanding of the underlying molecular mechanisms and using predictive biomarkers that can identify responsive tumors are essential to maximize therapeutic benefits while minimizing toxicity.

### 6.3. Prevention Strategies: Identifying High-Risk Individuals

Considering the consistent epidemiological evidence showing that metformin protects against CRC, its potential role in chemoprevention, particularly in high-risk groups, warrants further investigation [63]. Identifying individuals who would benefit the most from such preventive strategies could also involve biomarkers. These might include:

- High-risk individuals with diabetes: Using metformin in patients with diabetes who are at a high risk for CRC could reduce the incidence, building on existing observational data [50]. Biomarkers related to diabetes control or specific risk factors for CRC in such patients could render this approach more precise.
- In patients with adenomatous polyps, metformin may inhibit the formation or progression of precancerous lesions, which is a key step in CRC development [64]. Biomarkers associated with polyp progression could help to identify individuals who would benefit from metformin for chemoprevention.
- Chemoprevention of hereditary CRC syndromes: Investigating the potential of metformin in individuals with a genetic predisposition to CRC could offer a valuable preventive strategy [65]. The specific genetic mutation that causes this syndrome can serve as a criterion for selecting individuals for metformin chemoprevention.

Prospective studies are needed to confirm these preventive benefits and determine the optimal dose and duration of chemoprevention, ideally by incorporating biomarkers to identify the most suitable candidates.

## 7. Challenges and Future Directions: Prioritizing Biomarker Validation and Implementation

Despite promising data, several significant challenges remain in fully realizing the therapeutic potential of metformin in CRC and implementing effective personalized approaches based on biomarkers.

### 7.1. Current Limitations: Bridging the Gap to Personalized Clinical Use

The transition from the observed benefits of metformin from laboratory studies and epidemiological links to routine, personalized clinical practice faces several hurdles, particularly when considering metformin use outside patient groups with diabetes and advanced disease, to reliably predict outcomes.

- Optimal dosing, duration, and timing of metformin treatment: The optimal dose, duration, and timing of metformin treatment specifically for anticancer effects, which might differ somewhat from those used for blood sugar control, have not been definitively established in large prospective clinical trials designed with cancer outcomes as the primary goal [48].
- Efficacy in patients without diabetes and identification of responders: Much of the most convincing clinical evidence has been derived from studies involving individuals with diabetes [6,7]. Confirming the efficacy of metformin and identifying reliable predictive biomarkers of response in patients without diabetes are critical unmet needs that must be addressed to expand the clinical usefulness of metformin in CRC.
- Limited efficacy as monotherapy for advanced disease: Metformin is generally not considered a cure when used alone for advanced or metastatic CRC. Its main potential is prevention, early disease staging, and combination treatment. Appropriate biomarkers must be identified to select patients for specific applications [49].
- Variability and confounding factors in observational studies: Informative observational studies are subject to influencing factors and biases (such as immortal time bias) that can affect the results and contribute to differing findings. This makes it challenging to draw firm conclusions concerning the efficacy across all subgroups without validating the findings using biomarkers.
- Validation of predictive biomarkers: Promising biomarkers have been identified in laboratory studies and studies examining patient data; however, they require rigorous validation in separate, well-designed clinical trials before they can be routinely used to guide treatment decisions in a personalized manner.

### 7.2. Future Research Priorities: The Centrality of Biomarkers

Addressing these challenges and moving the field forward requires focused future research efforts that prioritize the role of biomarkers at every step.

- Prospective clinical trials with the required biomarker stratification: designing and undertaking large randomized controlled trials that mandate biomarker testing to divide patients into groups and assess the effectiveness of metformin within these clearly defined subgroups are essential. This approach will validate predictive markers and establish their efficacy in specific populations where the drug is most likely to work.
- Mechanistic studies linking differential responses to biomarkers: further research is needed to fully understand the molecular and cellular reasons why patients respond differently to metformin treatment. Future research should specifically investigate how these mechanisms are affected by and tied to the identified biomarkers (e.g., how *KRAS* mutations or MATE1 expression levels mechanistically determine sensitivity or resistance) [10].
- Development and validation of novel biomarkers: The continued exploration of new predictive biomarkers is crucial for improving the identification of responsive patients. This could involve markers identified in liquid biopsies, advanced imaging techniques, or comprehensive ‘multi-omics’ profiling (genomics, transcriptomics, proteomics, metabolomics).
- Biomarker-driven exploration of the effects of metformin in non-diabetic populations: dedicated prospective clinical trials are needed to evaluate the effectiveness and safety of metformin in preventing or treating CRC in individuals without diabetes who are at high risk or have been diagnosed. These trials should include integrated biomarker analysis to determine which patients without diabetes respond positively.
- Biomarker-guided exploration of novel combination strategies: laboratory and clinical studies investigating suitable combinations of metformin and chemotherapy, targeted therapies, immunotherapy, and other metabolic inhibitors should be guided by preclinical studies and validated biomarker profiles. This will help select patient populations that are most likely to benefit from a specific combination.
- Mechanistic studies of sex-specific differences and potential biomarkers: further research is required to understand the biological basis of the observed differences between the sexes in terms of how metformin affects CRC outcomes. This may involve hormonal or metabolic differences that could serve as biomarkers for tailoring treatment strategies based on sex [6].
- Advanced biomarker discovery technologies: emerging platforms, including multi-omics integration using machine learning [66], liquid biopsy for real-time monitoring [67], AI/machine learning for pattern recognition [68], single-cell sequencing for cellular heterogeneity analysis [69], and spatial transcriptomics for microenvironment mapping [70] offer new opportunities for biomarker discovery and validation by identifying complex signatures, tracking dynamic treatment responses, and providing mechanistic insights beyond traditional approaches.

## 8. Conclusions

Metformin, a long-standing and safe medication for diabetes, holds significant promise as a valuable agent for cancer prevention and treatment. While compelling data from population studies highlight its benefits, particularly in patients with diabetes, evidence also points to varying responses across a broader spectrum of patients with CRC [6,7]. This variation highlights that the key to fully harnessing the potential of metformin and making it a widespread clinical application lies in identifying specific patient groups that are most likely to benefit. Therefore, identifying predictive biomarkers, such as *KRAS* mutation status [10], MATE1 expression levels [10,16], and emerging metabolic or immune-related patterns [13,15], is of paramount importance in moving toward personalized CRC management.

Metformin has multiple mechanisms of action, affecting cell metabolism [19], signaling pathways [32,37], and the TME [52,55], providing a strong biologically based foundation for its anticancer effects and offering various targets for potentially synergistic combinations. The effectiveness of these combinations can be predicted by using biomarkers. Recent studies exploring new combinations of metformin with immune checkpoint [49] and metabolic inhibitors [18] have revealed an expanding range of potential treatment strategies for boosting the anticancer effects of metformin.

Although challenges remain regarding finding the optimal dose, demonstrating effectiveness in individuals without diabetes, and defining its exact role in advanced disease, the convergence of laboratory insights, population-level evidence, and increasing identification of predictive biomarkers strongly supports the continued investigation of metformin for CRC management. However, critical research gaps must be addressed: (1) the lack of validated biomarkers for non-diabetic populations that represent most patients with CRC [9]; (2) insufficient patient stratification methods, as evidenced by high heterogeneity across studies [6,7]; (3) suboptimal integration into treatment paradigms [49]; and (4) incomplete mechanistic understanding across different patient populations. Future research must prioritize well-designed clinical trials that mandate biomarker-based patient selection and the development of rational combination strategies guided by these biomarkers. Advanced technologies, including AI-driven biomarker discovery and single-cell analysis, are essential to address these gaps [68,69]. This approach is essential to maximize the therapeutic potential of this widely used and well-tolerated drug for treating CRC, ultimately moving toward a more precise and personalized approach to CRC therapy.

## Figures and Tables

**Table 1:** Key predictive biomarkers for metformin response in CRC.

Biomarker	Type	Proposed Mechanism	Predicted Response	Reference
KRAS Mutation Status	Genetic Mutation	Silencing of MATE1 expression in mutant KRAS tumors → Increased intracellular metformin accumulation	KRAS-mutant tumors are more sensitive to metformin	[10]
MATE1 (Multidrug and Toxic Compound Extrusion 1) Expression	Protein Expression	Silencing of MATE1 expression (via DNMT1-mediated hypermethylation) → Decreased metformin efflux → Increased intracellular metformin accumulation	Low MATE1 expression is associated with increased metformin efficacy	[10]
OCT and MATE2 Transporter Profiles	Protein Expression	Regulate intracellular metformin concentration (OCTs for uptake, MATEs for efflux)	High MATE2 expression may be associated with resistance. High OCT3 expression may be associated with increased sensitivity	[11]
AMPK Pathway Components	Protein Activity/Status	High basal AMPK activity increases sensitivity to metformin-induced growth inhibition	High AMPK activity is associated with increased metformin effects	[12]
Glutamine Metabolism	Metabolic Profile	Glutamine-dependent CRC cells show resistance to metformin	Glutamine dependency is associated with resistance	[13]
Transcriptomic Profiles	Gene Expression Pattern	Specific gene expression patterns, potentially reflecting metabolic or signaling signatures (e.g., COX11, immune-metabolic patterns), correlate with response	Specific gene expression patterns predict response	[14,15]

**Table 2:** Key clinical evidence on metformin in CRC.

Study Type	Patient Population	Key Findings (Metformin Effect)	Implication for Patient Stratification/Biomarkers	Reference
Meta-analyses & Systematic Reviews	CRC, particularly in patients with diabetes	Reduced CRC incidence and adenoma formation; improved overall survival and CRC-specific survival (especially in diabetic patients); Sex-specific differences in survival benefit (stronger in women).	Heterogeneity in effect sizes across studies/cohorts highlights the need for predictive biomarkers to select responsive patients.	[6,7]
Nationwide Cohort Studies	Patients with diabetes and post-diagnostic CRC	Metformin use after CRC diagnosis is associated with reduced all-cause mortality and CRC-specific mortality.	The observational nature limits definitive conclusions across all subgroups without biomarker validation. Highlights potential therapeutic role in diagnosed patients.	[48]
Clinical Trials (Phase II)	Treatment refractory microsatellite-stable metastatic CRC	Combination of nivolumab and metformin showed limited clinical efficacy; Correlative studies suggested potential immune-modulatory effects (e.g., increased tumor-infiltrating lymphocytes).	Limited response underscores the complexity of advanced disease and the critical need for better patient selection based on predictive biomarkers for combination therapies.	[49]

## Abbreviations

CRC	colorectal cancer
T2DM	type 2 diabetes mellitus
AMPK	AMP-activated protein kinase
mTOR	mammalian target of rapamycin
ROS	reactive oxygen species
TME	tumor microenvironment
